# Translation and validation of Persian version of sexual function: vaginal changes questionnaire (SVQ) for women with gynecologic cancers

**DOI:** 10.1186/s12905-022-01863-2

**Published:** 2022-07-08

**Authors:** Raziyeh Maasoumi, Farinaz Rahimi, Somayyeh Naghizadeh

**Affiliations:** 1 Nursing and Midwifery Care Research Center, School of Nursing and Midwifery, Tehran University of Medical Sciences, Tehran, Iran; 2 Department of Midwifery and Reproductive Health, School of Nursing and Midwifery, Tehran University of Medical Sciences, Tehran, Iran; 3 Department of Reproductive Health and Midwifery, Faculty of Medical Sciences, Tarbiat Modares University (TMU), Tehran, Iran; 4 Department of Midwifery, Faculty of Medical Sciences, Tabriz Medical Sciences, Islamic Azad University, Tabriz, Iran

**Keywords:** Gynecologic cancer, Sexual function, Vaginal function, Validity, Reliability

## Abstract

**Background:**

Changing in the sexual function is an important condition in women with gynecological cancers. A valid and reliable questionnaire is required to assess this condition. The aim of this study was to translate and validate the Persian version of the Sexual-Vaginal Function Changes Questionnaire (SVQ) in women with gynecologic cancers.

**Methods:**

This methodological study with a psychometric design was conducted on 250 women with gynecologic cancers, who visited public and private medical centers in Tehran to receive follow-up services. Convenience sampling was conducted from April 2019 to May 2020. First, the Persian version of the Sexual Function-Vaginal Changes Questionnaire was developed and then, psychometric properties such as content validity, face validity, construct validity and criterion validity were assessed. Reliability of the instrument was assessed by Cronbach's alpha coefficient.

**Results:**

Mean age of participants was 53.3 ± 11.8 and mean score of SVQ was 63.0 ± 9.1 in the possible range of 26–104. Content validity was tested through qualitative method and six items were revised as suggested by the expert panel. Cronbach's alpha coefficient was 0.71 for the whole questionnaire and it was 0.93, 0.92, 0.89, 0.78, 0.88 and 0.78 for the 6 subscales, respectively, which shows the optimal internal consistency. Results of exploratory factor analysis revealed six factors as (1) intemacy and sexual interst, (2) arousal, (3) changes in intemacy and sexual interst after cancer, (4) vaginal changes after cancer, (5) vaginal bleeding during coitus, (6) and sexual worry and dissatisfaction after cancer. explained 70.09% of the variance observed. Criterion validity test of the questionnaire showed a significant correlation between the total SVQ scores and the total Female Sexual Function Index (FSFI) scores as well as between SVQ dimensions and dimensions of FSFI (*P* < 0.001).

**Conclusions:**

The findings from this study indicated that the Persian version of the SVQ is a valid and reliable instrument to assess sexual function-vaginal changes in women with gynecologic cancers.

## Background

Gynecologic cancer (GC) is any cancer that starts in a woman's reproductive organs [1]. Gynecologic cancers account for 16% of female malignancies worldwide [2]. GC treatments include surgery, chemotherapy, radiotherapy, or combination therapy. With medical advances, the number of GC survivors has increased [3]. With improved effectiveness of cancer treatments, the number of GC survivors has been reported to exceed 1 million in 2016 [4].

The National Comprehensive Cancer Network (NCCN) provided guidelines for cancer survivors. In these guidelines for sexual function, health care providers are recommended to ask people about their sexual function at regular intervals [5]. Despite increased life expectancy in these women, gynecologic cancers may affect many aspects of women's health, including sexual function [6] and quality of life of GC survivors [3]. Sexual health is, therefore, considered as one of the components of quality of life and an important aspect in GC treatments [7, 8]. In their study, Olivia et al. revealed that women with ovarian cancer experience a lower quality of life and a higher rate of sexual dysfunction and distress [9]. Therefore, cancer and cancer treatments affect physical and mental function, which can significantly affect women's intimate relationship with their sex partner [10].

GC Survivors experience a wide range of sexual concerns following diagnosis and treatment [11, 12]. Sexual dysfunction is associated with treatment outcomes of surgery, radiation therapy, chemotherapy, and hormone therapy, as well as cancer itself [13, 14]. These women reported some problems including decreased sexual desire, vaginal dryness and/or dyspareunia, problems with sexual arousal and/or orgasm, and low sexual satisfaction [12, 15]. In addition to the physiological impacts, these women suffered from a wide range of psychological consequences such as loss of femininity, negative body image, and psychological distress [16]. Cancer and cancer treatment may even lead to a cessation of sexual activity [17]. In the US, Blake et al. (2017) conducted a study on women with GC using FSFI and revealed that women undergoing chemotherapy suffered from post-cancer treatment sexual dysfunction [18].

Sexual dysfunction is reported in most patients visiting for cancer treatment follow-up care; therefore, health care providers should evaluate their sexual performance [19]. Various tools have been used so far to assess women's sexual health such as Female Sexual Dysfunction Index (FSFI), Quality of Life—Cancer Survivors (QOL-Cs), Questionnaire on Screening for Sexual Dysfunction (QSD), and The Functional Assessment of Cancer Therapy-Endometrial (FACT-En). The FSFI measures general aspects of female sexual dysfunction; it has been developed and validated for normal women with sexual dysfunction and does not cover cancer and cancer treatment [20]. QOL-Cs assess various aspects of quality of life in cancer survivors; however, it covers all types of cancers [21] not just GC. QSD examines sexual dysfunction and different stages of sexual cycle [22]. None of these inventories specifically assess sexual function in women with GC. Sexual-Vaginal Function Changes Questionnaire (SVQ) was first developed and psychometrically tested by Jensen et al. (2004) in Denmark to assess post-GC treatment sexual function [20]. It was psychometrically tested by Chow et al. (2010) in China [7].

Cervical cancer, ovarian cancer and uterine cancer are the second, eighth and eleventh most common cancers among Iranian women. The first report by Iran Cancer Research Center indicated that the incidence of these cancers in Iran is 4.8, 4.6 and 2.6, respectively [23]. Given the high prevalence of these cancers among Iranian women and increased life expectancy in them due to the improved and effective cancer treatments, the number of Iranian GC survivors is increasing. Therefore, sexual complications and disorders following cancer treatments have also increased in these women [24]. To study these problems, a valid and reliable measure is required. Therefore, due to the lack of questionnaire and these problems associated GC survivors, the aim of this study was to translate and validate the Persian version of the Sexual-Vaginal Function Changes Questionnaire in Persian women with gynecologic cancers.

## Methods

### Type of study and participants

This methodological study with a psychometric design was conducted on women with gynecologic cancers using convenience sampling from April 2019 to May 2020. The research setting was 4 medical centers in Tehran (Be'sat and Loghman hospitals as a public centers and Roshana and Kimia oncology clinics as a private centers). Convenience sampling was used to select medical centers from different districts in Tehran.

Inclusion criteria were literacy for reading and writing, being married, and sexually active (having any type of sexual relationship, including a range of activities such as foreplay, penetration and afterplay) within the last 6 months. All of these criteria were provided using self-report. The other inclusion criteria were definitive diagnosis of endometrial, cervical or ovarian cancer and in a state of recovery period based on the medical records. Exclusion criteria were suffering from or the history of known mental illnesses that affect sexual function, experiencing an unfortunate event in the past 3 months before the start of the study (death of loved ones etc.) and having extramarital affairs of each couple. All of exclusion criteria were provided using self-report. It is necessary to explain that the data were collected using closed-ended questions when women's physical and mental conditions were stable. In order to estimate the sample size in this study, the sample size should be 10 times of the number of items in the instrument [25]. Thus, a sample of 240 was obtained and considering a 10% sample loss, a total sample size of 250 was calculated.

### Sampling

After receiving the code of ethics from the Joint Organizational Ethics Committee of the School of Nursing and Midwifery and the School of Rehabilitation of Tehran University of Medical Sciences (Code: IR.TUMS.FNM.REC.1397.208) and presenting a letter of introduction to the authorities of the relevant medical centers, the relevant permit was obtained for sampling. All eligible women with endometrial, cervical and ovarian cancers who were willing to participate entered the study and the objectives were explained to them. Before starting the study, the participants were provided with necessary explanations about the objectives and method of the study, voluntary participation, respect for privacy, confidentiality and the right to refrain from any data collection stage. As a compensation, the samples informed about that those who wished to receive sexual education or counseling about their sexual concerns or problems related to the GCs, they could be contact to the researcher team after participate in the study. They were assured that there was no need to write their first and last name and that all their information would remain confidential with the researcher. Informed written consent was obtained from participants. This study was conducted in two phases. First, the Persian version of SVQ was developed and then, it was psychometrically tested.

### Data collection tools

Three inventories, including demographic profile scale, SVQ and the FSFI were used to collect data.

#### Persian version of sexual function -vaginal changes questionnaire (SVQ)

Jensen et al. (2004) first developed and conducted psychometric testing on SVQ in Denmark [20] to investigate post-GC treatment sexual function. This questionnaire consists of 27 items and 5 subscales, including intimacy (IN), global sexual satisfaction (GS) and sexual interest (SI). All participants answered the scale regardless of having the spouse available or sexual function. Vaginal changes (VC) and sexual function (SF) were answered only by sexually active patients. Then, Chow et al. (2010) conducted psychometric testing of this scale in China [7]. Psychometric testing of the instrument in this study was based on the translation and adaptation process provided by the World Health Organization [26].

### Translation

After obtaining permission from the original developer of the questionnaire, it was translated into Persian in four stages. First, the questionnaire was Forward Translation by two Persian-speaking English translators. Then, the translated versions were compared by the expert panel in the presence of two translators and a common translation was agreed upon. In the third stage (backward translation), two English-speaking translators who were fluent in Persian and had not previously seen the English version of the questionnaire were asked to back-translate the Persian version of the questionnaire into English. In the last phase, Pre-testing and cognitive interviews were conducted to measure the clarity of translated version and incomprehensible items were marked and revised. The translated version was given to 10 applicants who met the inclusion criteria. Their views about each item were asked through personal interviews, and the items that were not understood were identified and the necessary changes were made. Finally, the final Persian version was approved.

### Validity

Content validity, face validity, construct validity and criterion validity were used to validate SVQ.

#### Content validity

Content validity was tested through qualitative method. In the qualitative content analysis, the author gave the questionnaire to 10 specialists in oncology, sexual and reproductive health, health promotion, health psychology and social medicine and the necessity, clarity and simplicity of the instrument were examined.

#### Face validity

To conduct face validity analysis, it was tried to adopt fluent writing style with appropriate wording. To this purpose, the translated questionnaire was presented to 15 eligible women and they were asked about the simplicity, comprehensibility and clarity of the items to evaluate qualitative face validity. To test quantitative face validity, a 5-point Likert scale was used (Very Important = 5, Important = 4, Moderately Important = 3, Slightly Important = 2, Not Important = 1) and the impact score was asked. Impact score of 1.5 or higher approved quantitative face validity.

#### Construct validity

Construct validity determines whether a questionnaire can meet the objectives of the study [27], and exploratory factor analysis indicates whether the instrument items are properly arranged. To measure construct validity, the correlation of the items before factor analysis should be examined using Bartlett's test and KMO index and this index should not be less than 0.5 [28]. To examine construct validity, exploratory factor analysis with orthogonal varimax rotation was used by maximum likelihood method in SPSS 22 with a factor loading greater than 1 and load factor larger than 0.4.

#### Criterion validity

The Persian version of FSFI was used in this study to test criterion validity of SVQ. To this purpose, both scales were given to participants and data were collected simultaneously. The Persian version of the standard FSFI developed by Rosen et al. [29] has 19 items in 6 dimensions, including sexual desire (2 items), arousal (4 items), lubrication (4 items), orgasm (3 items), sexual satisfaction (3 items) and pain (3 items).

### Reliability

To determine the reliability, the Cronbach's alpha coefficient was measured for the whole questionnaire and its subscales.

## Results

Mean ± standard deviation of women's age was 53.3 ± 11.8. Of participants, 28*%* had elementary education and only 20.4*%* had academic degrees. Two-third of participants (66.4*%*) were housewives. Mean ± standard deviation of length of marriage was 31.1 ± 16.4. Mean ± standard deviation of duration of cancer was 21.2 ± 20.6 months and nearly half of the participants (46.8*%*) were diagnosed with grade II cancer. Demographic data and clinical information of participants are shown in Table 1.

**Table 1:** Demographic characteristics and clinical information of the participants (n = 250)

Number (Percent)	Characteristic
Age (Year)	M ± SD = 53.3 ± 11.8
29–39	83 (33.2)
40–49	109 (43.6)
50–69	58 (23.2)
*Education*	
Elementary	70 (28.0)
Secondary	33 (13.2)
Diploma	96 (28.4)
University	51 (20.4)
*Job*	
Housewife	166 (66.4)
Working at home	43 (17.2)
Working outside the home	41 (16.4)
Husband’s age	M ± SD = 53.3 ± 11.8
30–50	91 (36.4)
51–70	113 (45.2)
71–90	46 (18.4)
*Husband’s education*	
Elementary	71 (28.4)
Secondary	56 (23.6)
Diploma	64 (25.6)
University	56 (22.4)
*Husband’s job*	
Unemployed	62 (24.8)
Employee	104 (41.6)
Employee	103 (41.2)
Duration of marriage (year)	M ± SD = 31.1 ± 16.4
10 ≥	30 (12.0)
11–30	106 (42.4)
31–50	73 (29.2)
50 <	41 (16.4)
*Contraception*	
Menopause	146 (58.4)
Withdrawal	29 (11.6)
Condom	46 (18.4)
Hysterectomy	29 (11.6)
Duration of cancer (Month)	M ± SD = 21.2 ± 20.6
12 >	114 (45.6)
12–24	74 (29.6)
25–36	28 (11.2)
36 <	34 (13.6)
*Grade of cancer*	
1	66 (26.4)
2	117 (46.8)
3	57 (22.8)
4	10 (4.0)
*Treatments received*	
Surgery	110 (44)
Chemotherapy	15 (6)
Radiotherapy	8 (3.2)
All three	27 (10.8)
Medication	21 (8.4)
Surgery with chemotherapy or radiation therapy	69 (27.6)

### Content validity

Content validity was tested through qualitative method and six items were revised as suggested by the expert panel in the qualitative content validity assessment.

### Face validity

Results of qualitative face validity assessment by end users of the questionnaire indicated the connection and fitness of the items with the subject under study, the correct understanding of the items by the respondents and the lack of ambiguity or difficulty in understanding the items. Results of qualitative face validity assessment showed an impact score larger than 1.5.

### Construct validity

Results of Kaiser–Meyer–Olkin (KMO) test showed the sampling adequacy for exploratory factor analysis (KMO = 0.868). Bartlett's test of sphericity with *X*_*2*_ = 6327.442, *p* < 0.001 indicated that the correlation between items is large enough to perform maximum likelihood. The results showed six factors explained 70.09*%* of the variance (Table 2). In the 26-item version, factor loadings were named as follows after rotation:

- Factor 1, intimacy and sexual interest, including 8 items (1, 2, 3, 5, 6, 15, 16 and 17)
- Factor 2, arousal, including 5 items (7, 11, 11a, 12 and 12a)
- Factor 3, changes in intimacy and sexual interest after cancer, including 4 items (18, 19, 20, and 21)
- Factor 4, vaginal changes after cancer, including 4 items (14, 22, 23, and 24)
- Factor 5, vaginal bleeding during coitus, including 2 item (13 and 13a)
- Factor 6, sexual worry and dissatisfaction after cancer, including 3 items (8, 9, 10)

**Table 2:** Eigenvalues and variance extracted with maximum likelihood method

Total Variance Explained									
Factor	Initial Eigenvalues			Extraction Sums of Squared Loadings			Rotation Sums of Squared Loadings		
	Total	% of Variance	Cumulative %	Total	% of Variance	Cumulative %	Total	% of Variance	Cumulative %
1	10.149	37.591	37.591	4.564	16.905	16.905	6.720	24.887	24.887
2	4.302	15.933	53.523	7.784	28.830	45.735	3.548	13.142	38.029
3	2.537	9.397	62.920	2.456	9.097	54.831	3.230	11.965	49.994
4	1.561	5.783	68.703	1.864	6.902	61.733	2.650	9.814	59.809
5	1.180	4.370	73.073	1.552	5.748	67.481	1.757	6.508	66.317
6	1.011	3.744	76.817	.705	2.612	70.093	1.020	3.777	70.093

Extraction Method: Maximum Likelihood

**Fig. 1:**
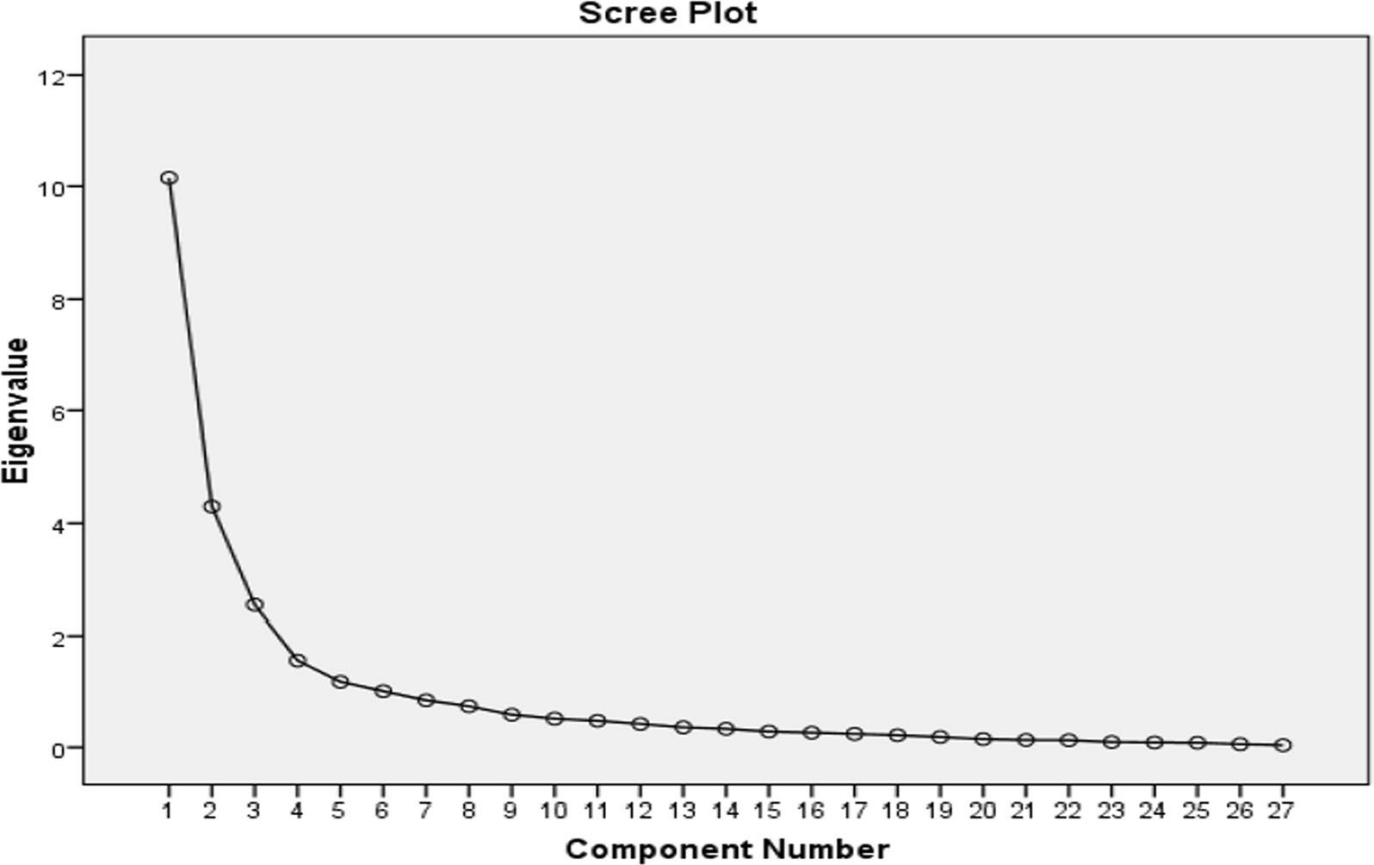
Scatter plot diagram of the distribution of factors identified in exploratory factor analysis

It is worth mentioning that item 14 was loaded on factors 2 and 4 and due to its compliance with item 2, it was moved there. Finally, 6 factors were explained, namely (1) intemacy and sexual interst, (2) arousal, (3) changes in intemacy and sexual interst after cancer, (4) vaginal changees after cancer, (5) vaginal bleeding during coitus, (6) and sexual worry and dissatisfaction after cancer The results of the structural validity based on maximum likelihood analysis are shown in Table 3.In adition, the distribution of factors explained by the exploratory factor analysis in the scree plot is shown in Fig. 1.

**Table 3:** Factor loading matrix for patterns identified in the SVQ questionnaire

Items	Factor loading					
	Factor1	Factor2	Factor3	Factor4	Factor5	Factor6
3. Have you had any interest in sexual relations?	**.881**	−.094	.237	−.040	−.057	−.066
16. Have you reached orgasm?	**.798**	−.302	.083	−.283	−.030	.055
9. How satisfied or dissatisfied have you been with your sex life/lack of sex life	**.764**	−.181	.196	−.110	−.102	**.482**
17. Did you feel relaxed after having sex?	**.757**	−.181	.053	−.191	−.140	.198
5. Has your partner wanted to have sexual relations?	**.754**	−.106	.005	−.117	−.026	.009
6. Have you had sexual relations?	**.738**	−.205	.068	−.042	−.007	.164
1. Have you been interested in close physical contact (a kiss and a cuddle)?	**.735**	−.240	.405	.110	−.044	−.138
10. How satisfied or dissatisfied have you been with your appearance?	**.718**	−.178	.192	−.166	−.062	**.503**
15. Were you able to complete sexual intercourse?	**.706**	−.264	.226	.039	−.047	−.119
2. Have you had close physical contact with your family and close friends?	**.675**	−.056	.312	.165	−.055	.022
7. Did your partner have difficulty achieving an erection?	**−.532**	**.373**	−.086	.005	−.026	.251
11a. If yes, has it bothered you?	−.317	**.851**	−.046	.183	.188	−.069
12a. If yes, has it bothered you?	−.263	**.847**	.003	.275	.159	−.084
11. Did you feel that your vagina was dry during intercourse?	−.388	**.794**	−.015	.258	.192	−.044
12. Have you had any pain during intercourse?	−.385	**.724**	.024	.362	.229	−.055
18. Has your interest in close physical contact changed since you were diagnosed with cancer?	.097	.026	**.961**	.016	.098	.008
19. How much close physical contact do you have with your family and close friends compared to before you were diagnosed with cancer?	.115	.021	**.915**	.080	.096	−.014
20. Has your interest in sexual relations changed since you were diagnosed with cancer?	.286	.078	**.766**	−.077	.063	.008
21. Has your partner’s interest in sexual relations changed since you were diagnosed with cancer?	.292	−.009	**.552**	−.333	−.061	.082
23. Do you feel that the size of your vagina has changed since you were diagnosed with cancer	.053	−.117	.024	**−.775**	−.083	.065
24. Has the pain you experience during intercourse changed since you were diagnosed with cancer?	−.039	.182	−.053	**.713**	.130	−.225
22. Has the dryness of your vagina changed compared to before you were diagnosed with cancer?	.082	.276	−.263	**.606**	.158	−.057
14. Did you feel that intercourse was bothersome because your vagina felt too small?	−.226	.368	.099	**.590**	.219	−.007
13. Have you experienced bleeding during intercourse?	−.125	.233	.106	.250	**.914**	−.133
13a. If yes, has it bothered you?	−.062	.327	.053	.252	**.750**	−.163
8. Has your sex life/lack of sex life made you worry?	−.039	.045	−.038	.228	.201	**−.487**

The symbol bold loading factors indicate the most significant number of factor loading in each dimension

### Criterion validity

Mean ± standard deviation of sexual—vaginal function changes (SVQ) was 62.9 ± 9.1 (in the possible range of 26–104), mean ± standard deviation of intimacy and sexual interest was 23.1 ± 6.2 (in the possible range of 8–41), arousal 10.5 ± 5.6 (in the possible range of 3–20), change in intimacy and sexual interest after cancer 7.5 ± 2.2 (in the possible range of 4–12), vaginal changes after cancer 7.9 ± 1.3 (in the possible range of 4–13), vaginal bleeding during coitus 2.4 ± 1.9 (in the possible range of 1–8), and Sexual worry and dissatifaction after cancer 11.3 ± 2.9 (in the possible range of 3–18). The Persian version of FSFI was used in this study to test criterion validity of SVQ. There was a significant correlation between total SVQ scores and total FSFI scores and as well as between SVQ dimensions and dimensions of FSFI (*P* < 0.001) (Table 4).

**Table 4:** Mean (SD) SVQ dimensions and its relationship with FSFI dimensions

Characteristic	Mean (SD)	Obtainable range	Earned range	Relationship with FSFI r (p)
1.Intimacy and sexual interest	23.1 (6.2)	8–41	11–33	0.703 (*P* < 0.001)
2.Arousal	10.5 (5.6)	3–20	3–20	0.511 (*P* < 0.001)
3.Change in intimacy and sexual interest after cancer	7.5 (2.2)	4–12	4–12	0.318 (*P* < 0.001)
4.Vaginal changes after cancer	7.9 (1.3)	4–13	5–11	0.220 (*P* < 0.001)
5.Vaginal bleeding during coitus	2.4 (1.9)	1–8	1–8	0.293 (P < 0.001)
6.Sexual worry and dissatifaction after cancer	11.3 (2.9)	3–18	3–17	0.625 (*P* < 0.001)
Sexual-Vaginal Function Changes (SVQ)	62.9 (9.1)	26–104	39–82	0.357 (*P* < 0.001)

### Reliability

Reliability of the instrument was assessed by Cronbach's alpha coefficient. Cronbach's alpha coefficient was 0.71 for the whole questionnaire and it was 0.93, 0.92, 0.89, 0.78, 0.88 and 0.78 for the 6 subscales, respectively, which shows the optimal internal consistency (Table 5).

**Table 5:** Reliability of the whole SVQ questionnaire and its subscales

	Number of items	Cronbach's alpha
1.Intimacy and sexual interest	8	0.93
2.Arousal	5	0.92
3.Change in intimacy and sexual interest after cancer	4	0.89
4.Vaginal changes after cancer	4	0.78
5.Vaginal bleeding during coitus	2	0.88
6.Sexual worry and dissatifaction after cancer	3	0.78
Sexual-Vaginal Function Changes (SVQ)	26	0.71

## Discussion

This study was conducted for the first time in Iran to translate and validate the Persian version of the sexual function—vaginal Changes Questionnaire (SVQ) for patients with GC. Validation of the Persian version of the SVQ proved it a valid and dedicated instrument to assess sexual function—vaginal changes in Iranian women with gynecologic cancers. Cronbach's alpha coefficient and internal consistency coefficient were acceptable with good reliability and stability for the questionnaire. In addition, there was reasonable qualitative content validity of this instrument.

SVQ was first developed and psychometrically tested by Jensen et al. in Denmark to assess post-GC treatment sexual function [20]. Results of their study found SVQ a reliable and valid scale [20]. Then, in 2010, Chow et al. conducted psychometric testing on this questionnaire and found SVQ a dedicated, reliable and applicable scale to assess sexual function in Chinese patients [7].

In this study, Cronbach's alpha coefficient was 0.71 for the Persian version of SVQ. Since it was larger than 0.7, internal consistency of the scale was approved. It was in the range of 0.78–0.93 for subscales which complied with the Cronbach's alpha coefficient obtained in the study by Jensen et al. for subscales (0.76–0.83) [20]. In the study by Chow et al. [7], internal consistency for the Chinese version of SVQ was 0.87 using Cronbach's alpha which shows its high validity. Cronbach's alpha coefficient was 0.64–0.88 for subscales which complied with this study.

The original SVQ consisted of two sections. The first section was for all patients (regardless of the spouse being available and sexual activity) with three subscales of intimacy, sexual desire and sexual satisfaction; the second section was for sexually-active patients with two more subscales in addition to the previous ones including vaginal changes and sexual function [20]. In the present study, given the principles of cultural adaptation and considering that sexual relations are legal in the context of marriage, being sexually active was considered as one of the inclusion criteria. Therefore, only sexually-active women were included in the study. The basis of translation and psychometric analysis of the Persian version of the SVQ was therefore the second structure of the main instrument proposed by Jensen et al. (2010) [20]; results revealed 6 factors as (1) intemacy and sexual interst, (2) arousal, (3) changes in intemacy and sexual interst after cancer, (4) vaginal changees after cancer, (5) vaginal bleeding during coitus, (6) and sexual worry and dissatisfaction after cancer.

The first dimension of the Persian version of SVQ consists of the questions related with the close physical contact, interest in sexual relations, the ability to complete sexual intercourse, and achievement of orgasm. The second dimension consists of the questions related with the genital condition during sexual function like as difficulty to achieve an erection in partner, vaginal dryness, pain during intercourse and feeling of bothering with dryness and pain. The third dimension consists of the questions related with the changes in close physical contact and interest in sexual relations after cancer. The fourth dimension consists of the questions related with the vaginal changes after cancer specially in size, dryness, and pain during intercourse. The fifth dimensions of the Persian version of SVQ consists of the questions related with the vaginal bleeding during coitus and feeling of bothering with its experience. Finally, the sixth dimension consists of the questions related with the worry and dissatisfaction about appearance and lack of sexual life after cancer.

Compering with the original version of SVQ, the first dimension of the Persian version can be explaining the first three dimensions of the original version of SVQ including intimacy (IN), global sexual satisfaction (GS), and sexual interest (SI). In addition, in the original version of SVQ, the dimensions of the vaginal changes (VC) and sexual function (SF) indicate all of changes in vagina and sexual function but in the Persian version, these dimensions were explored in the three constructs as arousal (the second dimension), vaginal changes after cancer (the fourth dimension), and vaginal bleeding during coitus (the fifth dimension). The last dimension of Persian version of SVQ can also be explain with the sexual interest area of the original version.

Comparing with the Chinese version of the SVQ and its Persian version showed that Chow et al.’s study dataset be inappropriate because the Kaiser–Meyer–Olkin (KMO) value was less than 0.6 but, in our study, the KMO value was more than 0.8. However, Chow et al.’s reported factor analysis supported the items applicable to sexually active respondents, clustered together into 2 hypothesized scales: vaginal changes (VC) and sexual function (SF) and they declared that their findings supported the results of the original study [7]. According to our results of the factor analysis, also, the dimensions of the original SVQ were supported with some changes that discussed above mentioned.

The results of criterion validity in the present study showed that the significant correlation between dimensions and total SVQ scores and FSFI. It could be supported that the basic assumption of the development of SVQ by Jensen et al. that they drafted SVQ from FSFI. In addition, these results indicated that SVQ can be a good alternative tool to the FSFI in the patents with GCs because SVQ can be measure the sexual function as well as FSFI and also shows the changes caused by GCs in female sexual function.

Results of the study found this questionnaire a valid and reliable instrument to conduct surveys in oncology settings on Iranian women population. Because cervical cancer, ovarian cancer and uterine cancer are the second, eighth and eleventh most common cancers among Iranian women [23]. Given the high prevalence of these cancers among Iranian women and increased life expectancy in them due to the improved and effective cancer treatments, the number of Iranian GC survivors is increasing. Therefore, sexual complications and disorders following cancer treatments have also increased in these women [24]. So, the Persian version of SVQ has the potential to be used as a practical tool in clinical practice and research for assessing sexual function problems in Iranian as well as non-Iranian but Persian language gynecological cancer patients, to identify those in need of appropriate counseling or other forms of intervention [19]. One of the limitations of this study was the potential bias in answering the questions due to the sensitivity of the research topic. It was tried to eliminate it by assuring the confidentiality of information and completing questionnaires anonymously. Although the present study confirms the psychometric properties of the Persian version of SVQ but to increase generalizability findings from this study are needed research should be conducted using this tool throughout similar setting. The future research should also be examining the confirmation of the factors of questionnaire between different GCs groups of respondents.

## Conclusion

The findings from this study indicated that the Persian version of the SVQ is a valid and reliable instrument to assess sexual function—vaginal changes in women with gynecologic cancers.

## Data Availability

Datasets used and analyzed during this study are available from the corresponding author on reasonable request.

## Abbreviations

SVQ: Sexual function and vaginal changes after gynecological cancer questionnaire
GC: Gynecologic cancer
NCCN: The National Comprehensive Cancer Network
FSFI: Female sexual function index
QOL-Cs: The quality of life-cancer survivors
QSD: Questionnaire on screening for sexual dysfunction
FACT-En: The functional assessment of cancer therapy-endometrial
IN: Intimacy
GS: Global sexual satisfaction
SI: Sexual interest
VC: Vaginal changes
SF: Sexual function
SD: Standard deviation
